# Pharmacogenetic Analysis of Pediatric Patients with Acute Lymphoblastic Leukemia: A Possible Association between Survival Rate and *ITPA* Polymorphism

**DOI:** 10.1371/journal.pone.0045558

**Published:** 2012-09-24

**Authors:** Hyery Kim, Hyoung Jin Kang, Hyo Jeong Kim, Mi Kyung Jang, Nam Hee Kim, Yongtaek Oh, Byoung-Don Han, Ji-Yeob Choi, Chul Woo Kim, Ji Won Lee, Kyung Duk Park, Hee Young Shin, Hyo Seop Ahn

**Affiliations:** 1 Department of Pediatrics, Cancer Research Institute, Seoul National University College of Medicine, Seoul, Korea; 2 Department of Pediatrics, Seoul National University College of Medicine, SMG-SNU Boramae Medical Center, Seoul, Korea; 3 YeBT Co., LTD, Seoul, Korea; 4 Department of Biomedical Sciences, Seoul National University College of Medicine, Seoul, Korea; 5 Department of Pathology, Seoul National University College of Medicine, Seoul, Korea; Indian Institute of Toxicology Reserach, India

## Abstract

Genetic polymorphisms are important factors in the effects and toxicity of chemotherapeutics. To analyze the pharmacogenetic and ethnic differences in chemotherapeutics, major genes implicated in the treatment of acute lymphoblastic leukemia (ALL) were analyzed. Eighteen loci of 16 genes in 100 patients with ALL were analyzed. The distribution of variant alleles were CYP3A4*1B (0%), CYP3A5*3 (0%), *GSTM1* (21%), *GSTP1* (21%), *GSTT1* (16%), *MDR1* exon 21 (77%), *MDR1* exon 26 (61%), *MTHFR* 677 (63%), *MTHFR* 1298 (29%), *NR3C1* 1088 (0%), *RFC1* 80 (68%), *TPMT* combined genotype (7%), *VDR* intron 8 (11%), *VDR* FokI (83%), *TYMS* enhancer repeat (22%) and *ITPA* 94 (30%). The frequencies of single nucleotide polymorphisms (SNPs) of 10 loci were statistically different from those in Western Caucasians. Dose percents (actual/planned dose) or toxicity of mercaptopurine and methotrexate were not related to any SNPs. Event free survival (EFS) rate was lower in *ITPA* variants, and *ITPA* 94 AC/AA variant genotypes were the only independent risk factor for lower EFS in multivariate analysis, which was a different pharmacogenetic implication from Western studies. This study is the first pharmacogenetic study in Korean pediatric ALL. Our result suggests that there are other possible pharmacogenetic factors besides *TPMT* or *ITPA* polymorphisms which influence the metabolism of mercaptopurine in Asian populations.

## Introduction

Over the past four decades, treatment of acute lymphoblastic leukemia (ALL) in children has improved dramatically [1]. This success is largely due to the decades of collaborative multicenter clinical trials which composed of combination drug therapy and risk stratification. Despite this success, drug resistance and treatment failure due to treatment related toxicities still occur in about 20% of patients [1].

One of the explanations of drug resistance and toxicities is the pharmacogenetic effect. Clinical observations of inherited differences in drug effects were first documented in the 1950s, giving rise to the field of pharmacogenomics, which uses genome-wide approaches to explain the inherited basis of differences between people in their response to drugs [2]. Germline polymorphisms in genes that code for proteins involved in the pharmacokinetics and pharmacodynamics of antileukemic agents are various, and inter-patient variability is the main factor for pharmacogenetic difference.

The germline polymorphisms in patients with ALL can alter drug metabolizing enzymes, drug transporters, or drug targets and thus influence the efficacy or toxicity of antileukemic agents. As a result, if the determinants of inter-patient variability in drug pharmacokinetics were better defined, individualized therapy based on those factors might solve drug resistance so that outcome is improved.

Since multiple chemotherapeutic agents are involved in treating ALL, many genes related to the metabolic pathways of those drugs have an effect on the pharmacokinetics of patients with ALL. In Korea, pharmacogenetic study including multiple genetic loci for pediatric ALL has not been reported.

In this study, the distribution of genetic polymorphisms and genes related to antileukemic drugs were analyzed, and their relations to the outcome of treatment and relapse rates were assessed. In addition, according to the institutional experience in the treatment of ALL, many patients could not tolerate full dosages of Western protocols. The differences in the frequencies of mutant alleles of various genes related to different diseases have been reported [3], [4]. To determine the ethnic difference in pharmacogenetics, the incidence of variant alleles were compared with Western data throughout this study.

## Methods

### Ethics Statement

This study was approved by the Institutional Review Board of Seoul National University Hospital (H-0611-021-189). Informed written consents for blood sampling, collection, DNA analysis and review of their medical records were obtained.

### Patients and treatment

Of the patients who were diagnosed with ALL from October 1989 to April 2005 in Seoul National University Hospital (SNUH), 100 patients whose informed consents and samples were available were included. Peripheral blood samples at complete remission from the patients were analyzed for this study. Patients were assigned to the standard-risk group if the leukocyte count was less than 50×10^9^/L, and the age was 1 to 9 years at diagnosis. Otherwise, patients were assigned to a high-risk group. Patients with L3 phenotype were treated with protocols for Burkitt leukemia, and they were not included in this study. In the standard-risk patients, the treatment protocols were modified from the Children's Cancer Group (CCG)-1881 [5], 1891 [6] or CCG-1952 [7] protocols. The original CCG-1881 regimen consisted of induction, consolidation, interim maintenance, single delayed intensification and maintenance. CCG-1891 regimen increased delayed intensification from single to double. CCG-1952 consisted of intrathecal triple (methotrexate, hydrocortisone, cytarabine) instead of intrathecal methotrexate compared to the pre-existing regimens [8]. The protocol for high-risk patients was CCG-1882, which employed longer and stronger post induction intensification in patients with slow early response during induction.

Induction was started with initial risk group based regimens in all patients, and consolidation regimen was switched to a modified protocol from the CCG-1882 [9] in patients whose leukemic blasts of bone marrow on day 7 of induction were greater than 25%. If leukemic blasts on day 14 were still greater than 25%, the protocol used for the high-risk patients was restarted. If a patient had one or more of the following: a leukocyte count of at least 200×10^9^/L, hypodiploidy, age younger than 1 year, presence of t(9;22), or the 11q23 rearrangement, the patients proceeded to undergo hematopoietic stem cell transplantation when an appropriate donor was available.

The modification of all the protocols in our study was made from our institutional experience that many patients who had been given the same dose with the original CCG protocol had to stop or delay their chemotherapy due to moderate to severe toxicities. Thus, our institution made modifications of the original dose, so that the dose of mercaptopurine (6-MP) was reduced from 75 mg/m^2^ to 50 mg/m^2^ [10].

The medical records of 100 patients were retrospectively analyzed for the clinical data and history. Events were defined as any relapse, death, or secondary malignancies.

### Genotyping

DNA was extracted from normal blood cells in peripheral blood, which was collected in remission states. Candidate genes were selected by considering the previous clinical studies in which exhibited polymorphisms and encoded proteins of multiple genes involved in the pharmacokinetics or pharmacodynamics of antileukemic agents were described. Because there was no large scale comparative study which analyzed pharmacogenetic differences between Western and Asian patients, we selected most of SNPs from two large scale comprehensive Western studies [11], [12]. Cytochrome P4503A4 (*CYP3A4*) and cytochrome P4503A5 (*CYP3A5*) are involved in the metabolism of prednisolone, dexamethasone, vincristine, etoposide, and cyclophosphamide [13]. Glutathione-*S-*transferases (*GSTM1*, *GSTP1*, and *GSTT1*) metabolize the parent drug or metabolites of steroids, vincristine, anthracycline, methotrexate, cyclophosphamide, and etoposide [12]. Multi-drug resistance-1 gene (*MDR1*) encodes for P-glycoprotein, which belongs to the membrane transporter and functions as an efflux pump. Substrates of P-glycoprotein include anthracycline, vincristine, etoposide, and cyclophosphamide. The nuclear vitamin D receptor (*VDR*) belongs to the superfamily of nuclear hormone receptor [14].Binding of ligands to the VDR up-regulates the expression of *CYP3A*. Thiopurine methyl-transferase (*TPMT*) and inosine triphosphate pyrophosphatase (*ITPA*) are involved in the metabolism of 6-MP [10], [15]. Methotrexate enters cells by active transport via the reduced folate carrier (*RFC*) and interacts with methylene-tetrahydrofolate reductase (*MTHFR*) and thymidylate synthetase (*TYMS*) [16]. The *TYMS* gene has a unique tandem repeat sequence in the enhancer region that has been shown to be polymorphic, containing either two (*2R*) or three (*3R*) 28-bp repeats [17]. Glucocorticosteroids enter cells by passive diffusion and bind to and activate the glucocorticoid receptor (*NR3C1*) [18].

Genetic analysis was performed using both conventional and multiplex polymerase chain reaction (PCR) methods. We developed a novel specific bulging specific (SBS) primer that reduced bias in the identification of leukemia-specific fusion gene transcripts and a multiplex amplification method using those SBS primers to amplify polymorphisms and termed it TotalPlex amplification [19]. Sixteen single nucleotide polymorphisms (SNPs) analyzed by TotalPlex amplification and SNP genotyping were *GSTM1* deletion, *GSTT1* deletion, *GSTP1* 313 A>G, *VD*R FokI (start-site) T>C, CYP3A5*3 (G>A at position 22,893), CYP3A4*1B (A>G at position_392), *TPMT* 238 G>C, *TPMT* 460 G>A, *TPMT* 719 A>G, *MTHFR* 677 C>T, *MTHFR* 1298 A>C, *RFC1* 80, *MDR1* exon 21 (2677 G>T/A), *MDR1* exon 26 (3435 C>T), *NR3C1* 1088 A>G, and *VDR* intron 8 G>A. Two SNPs were analyzed by uniplex PCR. *TYMS* enhancer repeats and *ITPA* 94 C>A were determined using PCR-restriction fragment length polymorphism analysis, as described previously [20], [21].

### Toxicity, dose modification, and genotypes

Toxicity of antileukemic agents could be higher in patients with specific genetic loci due to the alteration of metabolism. When hematologic or hepatic toxicity developed during the treatment, the dosage of the drug was reduced according to the guidelines of original CCG protocols. Therefore, toxicity was supposed to be in inverse proportion to the actual administered dosage of the antileukemic agent. Analysis was conducted with the percentage of the actual administered dose to the planned dose (dose percent). The actual administered doses of 6-MP and methotrexate (MTX) were investigated, and the dose percent of each drug was calculated. Mercaptopurine and MTX were administered regularly in maintenance schedules of CCG protocols, and toxicities were most likely to occur in maintenance schedules if a patient had a susceptible genotypic trait for low metabolism. Therefore, the doses of 6-MP and MTX in the final maintenance cycle were supposed to be the maximum tolerated doses for patients. The dose percents were calculated with the actual administered doses of the last maintenance cycle. To grade complications, the National Cancer Institute Common Toxicity Criteria (NCI-CTC) version 4.0 was used.

### Statistical analysis

The Hardy-Weinberg equilibrium was assessed by the chi-square test with df = 1 for all tested SNPs, except in *GSTM1* and *GSTT1* because there was no distribution of null or non-null heterozygote in both genotypes [22]. Pairwise linkage disequilibrium was analyzed using SNAP (http://www.broad.mit.edu/mpg/snap) among SNPs within same chromosome based on a phased genotype data from 1000 Genomes Pilot 1 in Asian analysis panel (CHB+JPT). The differences in genetic polymorphism between risk groups (high vs. standard) and other populations (Korean vs. Western or Japanese) were analyzed using the chi-square test or Fisher's exact test (when expected cell counts of less than 5 comprise 20% of more of two-by-two contingency tables). Event-free survival (EFS) and overall survival (OS) were estimated using Kaplan-Meier analysis, and the survival differences according to different genetic polymorphisms and prognostic variables were analyzed by log-rank test. Multivariate analysis was conducted with Cox proportional hazards regression model to analyze predictive factors. For the multivariate analysis, variables with *P*-value≤0.25 from univariate analysis were used as variables for multiple logistic regression analysis. All significant univariate variables were entered in a stepwise, forward-selection protocol. To analyze gene-gene interactions, the multifactor dimensionality reduction (MDR) analysis was performed using polymorphisms and presence or absence of event or death. The MDR analysis was performed using the open-source MDR software package (v.1.1.0; available at: http://www.epistasis.org). The dose percentages was divided by each genotype of specific locus, and comparison of the administered dose percentage according to each SNP group was analyzed by T-test or ANOVA for parametric data and Mann-Whitney U or Kruskal-Wallis test for nonparametric data. Correlation between the administered dosage percent of the drugs was done using linear regression analysis. SPSS version 19.0 was used for all statistical analyses, and statistical significance was accepted for *P*<0.05.

## Results

### Patients and treatment

A total of 100 patients were evaluated (Table 1). There were 57 males and 43 females. The median follow up duration was 105.2 months (1.7∼204.9 months). The median age of diagnosis was 5.2 years (1.4∼16 years). Sixty-nine patients were assigned to the standard-risk group, and 2, 12, and 55 patients were treated with modified CCG-1881, 1891, and 1952 protocols, respectively. Thirty-one patients were assigned to the high-risk group and treated with the modified CCG-1882 protocol. Ninety-two patients had precursor B-cell immunophenotype and six patients showed precursor T-cell lineage. A frequent translocation identified by conventional chromosomal analysis, FISH examination, or both was t(12;21) in 17 patients. The number of patients with hyperdiploidy (≥50) and hypodiploidy (<44) were 12 and 4, respectively. Six patients received allogeneic hematopoietic stem cell transplantation (HSCT) from unrelated donors (5 double units cord blood, 1 peripheral blood stem cell) at the second complete remission (CR), and 1 patient with Philadelphia chromosome underwent autologous hematopoietic stem cell transplantation at the first CR.

**Table 1:** Patient characteristics (N = 100 patients).

Characteristics	
**Age (yr), median (range)**	5.2 (1.4–16)
1 y to less than 10 y	80
at least 10 y	20
**Gender, No.**	
Male	57
Female	43
**WBC count at diagnosis, No.**	
<10,000/µL	61
10,000/µL–100,000/µL	35
>100,000/µL	3
>200,000/µL	1
**Risk group, No.**	
Standard-risk patients	69
modified CCG-1881	2
modified CCG-1891	12
modified CCG-1952	55
High-risk patients	
modified CCG-1882	31
**FAB classification, No.**	
L1	75
L2	19
Not identified	6
**Immunophenotype, No.**	
Precursor B	92
Precursor T	6
Not available	2
**Cytogenetics, No.**	
t(12;21);TEL-AML1	17
t(9;22);BCR-ABL1	2
t(1;19);E2A-PBX1	1
p16 deletion	2
hyperploidy	12
hypoploidy	4
Complex karyotype	4
Normal karyotype	44
Down syndrome (21 trisomy)	2
Others	7
Not available	5
**CNS involvement, No.**	
Absent	89
Present	5
Not available	6
**Day 7 BM response to treatment, No.**	
Leukemic blasts less than 25%	73
Leukemic blasts at least 25%	27
**Event**	
Relapse	12
BM	4
CNS	3
Testis	2
BM+CMS	2
BM+testis	1
Death	4
Secondary malignancy	0
**Grade 3,4 toxicity during treatment**	
Hyperbilirubinemia	1
Liver enzyme dysfunction	23
Febrile neutropenia without sepsis	13
Sepsis	15

Abbreviation: CCG, Children's Cancer Group.; BM, bone marrow; CNS, central nervous system.

### Pharmacogenetic analysis

Genotypes of the 18 candidate loci in 100 patients are summarized in Table 2. The distribution of variant alleles were CYP3A4*1B (0%), CYP3A5*3 (0%), *GSTM1* (21%), *GSTP1* (21%), *GSTT1* (16%), *MDR1* exon 21 (77%), *MDR1* exon 26 (61%), *MTHFR* 677 (63%), *MTHFR* 1298 (29%), *NR3C1* 1088 (0%), *RFC1* 80 (68%), *TPMT* combined genotype (7%), *VDR* intron 8 (11%), *VDR* FokI (83%), *TYMS* enhancer repeat (22%), and *ITPA* 94 (30%). Each genotype was classified by risk group, and there was no difference in the distribution of variant alleles of all candidate genes in both risk groups. The estimation of Hardy–Weinberg equilibrium was not available in CYP3A4*1B A>G, CYP3A5*3 G>A and *NR3C1* 1088 A>G, because there was no patient with variant genotypes. The other genotypes were in Hardy–Weinberg equilibrium (*P*>0.05), except in *VDR* intron 8 G>A (*P* = 0.00) and *MDR1* exon 21 G>T/A (*P* = 0.002) (Table 2). In the case of *MDR1* exon 21 G>T/A, Hardy-Weinberg equilibrium was reached after the A variant was excluded (*P* = 0.38). This might suggest an effect of a recent mutation not yet reaching Hardy-Weinberg equilibrium, and the small sample size could lead to biased Hardy-Weinberg equilibrium estimations. Moreover, the bias or low *P* values have also been reported in previous studies which showed functional effects of the variants [23], [24]. Therefore, we included *MDR1* exon 21 G>T/A in analyses so that only *VDR* intron 8 G>A was excluded in the further analyses.

**Table 2:** The frequencies of candidate genetic loci.

			Total Enrolled		Western^a^		Japanese^b^		Risk group		
Loci	Genotypes	HWE *P*	(N = 100)	(%)	%	*P* a	%	*P* b	High	Standard	*P*
									(N = 31)	(N = 69)	
CYP3A4*1B ** A>G**	AA	NA	100	(100)	96.7	0.11	100	1	32	68	1
	AG/GG		0	(0)	3.3		0		0	0	
CYP3A5*3 ** G>A**	GG	NA	100	(100)	82.4	0.00	48.9	0.00	32	68	1
	AG/AA		0	(0)	17.6		51.1		0	0	
***GSTM1*** ** deletion**	Non-null	NA	79	(79)	53.9	0.00	NA		27	52	0.44
	Null		21	(21)	46.1				5	16	
***GSTP1*** ** 313 A>G**	AA/AG	0.24	79/21	(100)	90.1	0.00	98.8	1	32	68	1
	GG		0	(0)	9.9		1.2		0	0	
***GSTT1*** ** deletion**	Non-null	NA	84	(84)	82.4	0.85	NA		29	55	0.26
	Null		16	(16)	17.6				3	13	
***MDR1*** ** exon 21 G>T/A**	GG	0.002	23	(23)	20.9	0.73	23.3	1	6	16	0.79
	AA/GA/GT/TT/TA	(0.38^c^)	3/13/36/9/16	(77)	79.1		76.7		24	48	
***MDR1*** ** exon 26 C>T**	CC	0.26	39	(39)	15.4	0.00	25.6	0.06	12	27	1
	CT/TT		51/10	(61)	84.6		74.4		20	41	
***MTHFR*** ** 677 C>T**	CC	0.54	37	(37)	44	0.38	39.5	0.82	12	25	1
	CT/TT		50/13	(63)	56		60.4		20	43	
***MTHFR*** ** 1298 A>C**	AA	0.76	71	(71)	53.9	0.02	66.7	0.61	21	50	0.48
	AC/CC		27/2	(29)	46.1		33.3		11	18	
***NR3C1*** ** 1088 A>G**	AA	NA	100	(100)	94.5	0.02	83.7	0.00	32	68	1
	AG		0	(0)	5.5		16.3		0	0	
***RFC*** ** 80 A>G**	AA/AG	0.80	32/48	(80)	63.7	0.02	80.2	0.89	24	56	0.43
	GG		20	(20)	36.3		19.8		8	12	
***TPMT*** ** genotypes** d	*1/*1	0.72	93	(93)	92.3	0.78	NA	0.58	32	61	0.09
	*1/*3A//*1/*3C//*2/*2		1//5//1	(7)	7.7				0	7	
***VDR*** ** intron 8 G>A**	GG	0	89	(89)	42.9	0.00	75.6	0.02	30	59	0.50
	GA/AA		3/8	(11)	57.1		24.4		2	9	
***VDR*** ** Fokl T>C**	TT/TC	0.94	17/48	(65)	59.3	0.46	76.5	0.12	21	44	1
	CC		35	(35)	40.7		23.5		11	24	
***TYMS*** ** enhancer repeat**	3R/3R	0.19	78	(78)	25.3	0.00	NA		20	50	1
	2R/3R//2R/2R		19//3	(22)	74.7				6	15	
***ITPA*** ** 94 C>A**	CC	0.68	70	(70)	78.4	0.06	80	0.14	22	48	1
	AC/AA		28/2	(30)	21.6		20		10	20	

The most common allele for each locus is underlined.

Abbreviation: HWE, Hardy–Weinberg equilibrium.

a Comparison between our data and Western data with pediatric ALL. Data for 15 loci was adopted from Ref. 10 and data of *ITPA* 94 C>A was from Ref. 9.

b Comparison between our data and normal Japanese data. Reference data was adopted by SNP searching on NCBI reference assembly (http://www.ncbi.nlm.nih.gov/snp/). Data for CYP3A4*1B was from Coriell Cell Repository samples and all the others were from Japanese data of the Hapmap project.

c Hardy-Weinberg equilibrium was reached after the A variant was excluded (*P* = 0.38).

d Variant alleles : TPMT*1 (Wild type), TPMT*2 (238 G>C), TPMT*3A (460 G>A and 719 A>G), TPMT*3B (460 G>A), TPMT*3C (719 A>G).

The pairwise *r^2^* was 0.00 between SPNs on *CYP3A4* or *CYP3A5* and *MDR1*, and between *GSTM1* and *MTHFR*. These results showed no correlation among SNPs within same chromosome.by linkage disequilibrium analysis.

### Ethnic difference

The distribution of the mutant alleles in our study was compared to data of Western Caucasians (Table 2). Data of Western Caucasians for 15 loci (CYP3A4*1B A>G, CYP3A5*3 G>A, *GSTM1* deletion, *GSTP1* 313 A>G, *GSTT1* deletion, *MDR1* exon 21 G>T/A, *MDR1* exon 26 C>T, *MTHFR* 677 C>T, *MTHFR* 1298 A>C, *NR3C1* A>G, *RFC1* 80 A>G, *TPMT* combined genotype, *VDR* intron 8 G>A, *VD*R FokI T>C, and *TYMS* enhancer repeat) was adopted from historical control of Ref. 10, and data of *ITPA* 94 C>A was from Ref. 9. Rocha *et al.* (Ref. 10) conducted a pharmacogenetic study in 246 pediatric patients with ALL who were enrolled for St Jude Children's Research Hospital Total XIIIB study, and Stocco *et al.* (Ref. 9) investigated the effects of *ITPA* 94 C>A variants in 244 patients who were enrolled for the same protocol. As a result, a difference was noted in CYP3A5*3 G>A, *GSTM1* deletion, *GSTP1* 313 A>G, *MDR1* exon 26 C>T, *MTHFR* 1298 A>C, *NR3C1* A>G, *RFC1* 80 A>G, *VDR* intron 8 G>A, *TYMS* enhancer repeat, and *ITPA* 94 C>A.

Our data was compared with data of normal Japanese which was adopted by SNP searching on NCBI reference assembly (http://www.ncbi.nlm.nih.gov/snp/) (Table 2). Data of 12 loci was available, and statistical difference was significant only in 3 loci (CYP3A4*1B A>G, *NR3C1* A>G, and *VDR* intron 8 G>A).

### Toxicity, dose modification of 6-MP, MTX, and genotypes

**Figure 1:**
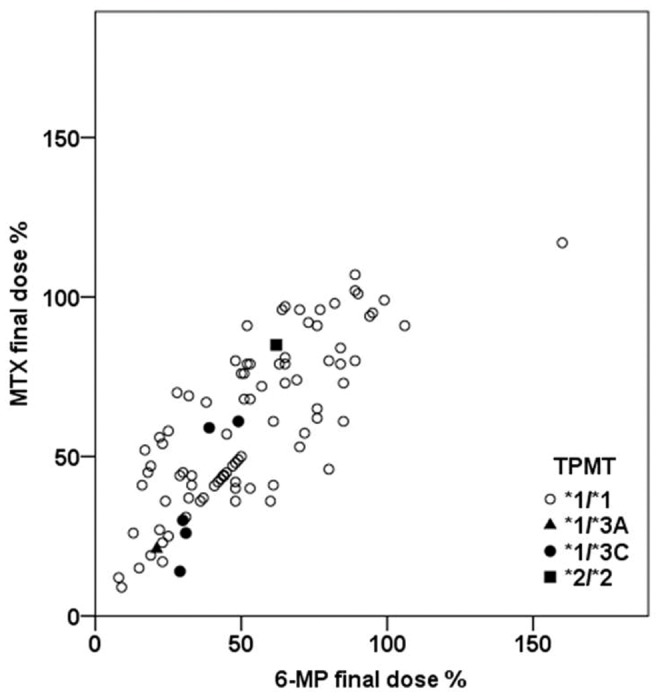
Correlation between the dose percent of 6-MP and MTX in each patient and *TPMP* genotype. The correlation analysis between the dose percent of 6-MP and MTX in each patient showed a statistically significant linear relationship (*R* ^2^ = 0.628, *P* = 0.00).

The median dose percents of 6-MP and MTX of the final maintenance cycle were 48.5% and 56.5%, respectively. Table 3 shows the distribution of dose percentage of 6-MP and MTX in the final maintenance cycle. Only 26/100 (26%) and 35/100 (35%) patients tolerated more than 75% of planned 6-MP and MTX doses, respectively. The dose percent was classified by *TPMT*. (Table 4). There was one patient with *TPMT* variant homozygote, and dose of the patient was not the lowest. However, the median dose percent of 6-MP and MTX for the *TPMT* wild type was higher than variant types. There was no significant difference in the dose percents by *TPMT* genotypes. The correlation analysis between the dose percent of 6-MP and MTX in each patient showed a statistically significant linear relationship (*R* ^2^ = 0.628, *P* = 0.00) (Figure 1). The analysis was also conducted between those dose percents and SNPs other than *TPMT*, and there was no notable correlation between the dose percent of 6-MP or MTX and the variant alleles of each candidate gene in our study.

**Table 3:** The distribution of dose percentage of 6-MP and MTX of the last maintenance chemotherapy.

Dose %	No. of patients	
	6-MP	MTX
<25	16	8
25–49	33	34
50–74	25	23
≥75	26	35

(*N* = 100 patients).

**Table 4:** The dose percentage of 6-MP and MTX of the last maintenance chemotherapy cycle by *TPMT* genotypes.

Genotype (No. patients)		Median dose % (range)					
		6-MP		*P*	MTX		*P*
	*1/*1 (93)	50	(8–160)		57	(9–117)	
*TPMT* genotype	*1/*3A, *1/*3C (6)	30.5	(21–49)	0.19	28	(14–61)	0.05
	*2/*2 (1)	62			85		

The wild type genotype is underlined.

Grade 4 sepsis occurred in 17 patients during total treatment. During maintenance treatment, grade 3,4 hyperbilirubinemia and grade 3,4 liver enzyme dysfunction occurred in 1 and 23 patients, respectively. Grade 3,4 febrile neutropenia during maintenance was observed in 13 patients. At the end of maintenance therapy, the estimated cumulative incidence of febrile neutropenia was 28.7%, and the cumulative incidence of febrile neutropenia was 27.2% in the *ITPA* wild type and 26.4% in the *ITPA* variant type (*P* = 0.242). There was no genetic locus related to risk of those complications.

### Survival analysis and genotypes

**Figure 2:**
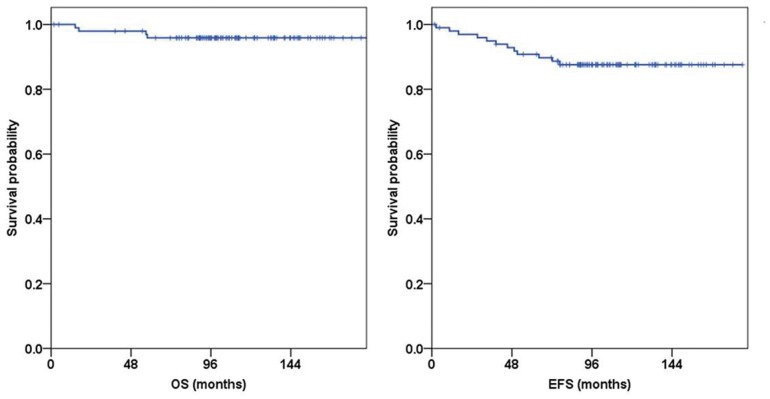
Overall survival and event free survival rates (*N* = 100). Estimated 10-year OS rate was 95.9% and EFS rate was 87.6% in 100 patients.

The estimated 10-year OS rate was 95.9%, and the EFS rate was 87.6% in 100 patients (Figure 2). A total of 5 patients had one or more very high-risk factors (1 patient: initial leukocyte count of at least 200×10^9^/L and hypodiploidy, 2 patients: presence of t(9;22), 2 patients: hypodiploidy).

A total of 12 patients relapsed during median follow-up duration of 105 months. Among those, 1 patient died of relapsed disease after autologous transplantation, and 3 patients died of treatment-related complications. The causes of treatment related mortality were sepsis (N = 1), veno-occlusive disease after HSCT (N = 1), and cytomegalovirus pneumonia after HSCT (N = 1). The details of relapsed sites were as follows: bone marrow (BM) (N = 4), central nervous system (CNS) (N = 3), testis (N = 2), BM and CNS (N = 2), and BM and testis (N = 1). There was no secondary malignancy reported.

**Figure 3:**
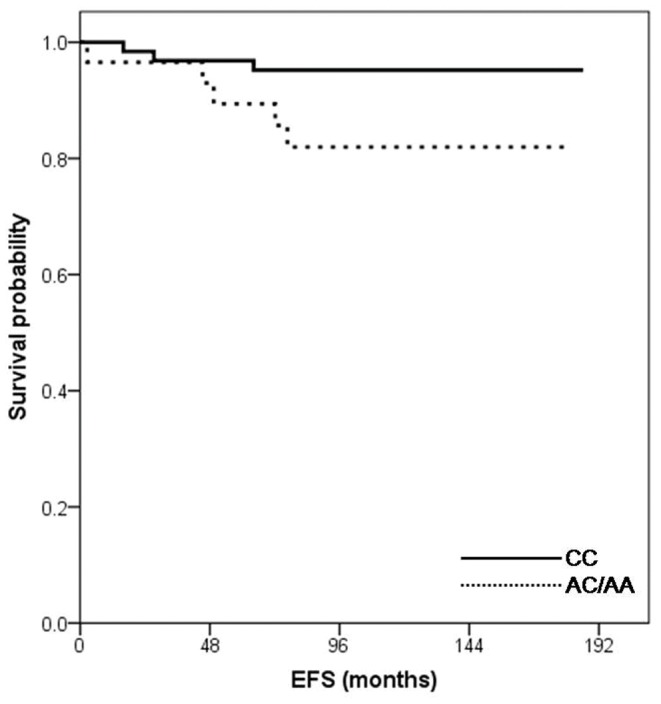
Overall survival and event free survival rate by *ITPA* 94 C>A genotypes (*N* = 95). *ITPA* 94 AC/AA genotypes were the independent risk factor for lower EFS. Event free survival rates were 95.2% in wild type, and 81.9% in AC/AA variant genotypes (*P* = 0.045).

Survival analysis with multiple variables was conducted in patients without very high-risk factors (N = 95). In the univariate analysis, the OS and EFS by age groups (1–9 yr vs.≥10 yr), gender, WBC counts at diagnosis, risk groups, chemotherapy regimens, FAB classification, CNS involvement, day 7 BM response and occurence of each grade 3,4 toxicity were not statistically different by each subgroup. We performed log rank analysis using genotypes of 12 loci which showed variant allele frequencies in this study (*GSTM1*, *GSTP1*, *MDR exon 21*, *MDR exon 26*, *MTHFR 677*, *MTHFR 1298*, *RFC 80*, *VDR intron 8*, *VDR fokI*, *TYMS*, *TPMT* and *ITPA*). Only statistically significant data was that EFS was lower in the *ITPA* 94 AC/AA variant genotypes in the univariate analysis (Figure 3). As a result of subsequent multivariate analysis with variables of marked differences in log rank tests (*P*<0.2), *ITPA* 94 AC/AA variant genotypes were the only risk factor for lower EFS (Table 5). Event free survival rate was 95.2% in wild type, and 81.9% in AC/AA variants (HR 4.96, 95% CI 1.1–22.7; *P* = 0.039) (Figure 3).

**Table 5:** Statistical results of analysis between survival rates and risk variables excluding patients with very high risk factors.

			Univariate		Multivariate	
Survival rate	Variables		(Log rank)		(Cox regression)	
	(No. of Events/Patients)		Survival rate	*P*	Hazard ratio	*P*
			(%)		(95% CI)	
**Overall survival**	*ITPA* 94	CC (1/65)	98.4	0.173		
		AC/AA (2/30)	92.6			
**Event free survival**	*ITPA* 94	CC (3/65)	95.2	0.045	4.96	0.039
		AC/AA (5/30)	81.9		(1.1–22.7)	
	*GSTT1*	Non-null (8/79)	89.3	0.189		
		Null (0/16)	100			
	*GSTM1*	Non-null (8/75)	88.7	0.132		
		Null (0/20)	100			
	*TPMT* genotype	3R/3R (5/73)	92.7	0.092		
		2R/3R (2/19)	89.5			
		2R/2R (1/3)	66.7			
	Sepsis	Yes (5/79)	93.4	0.107		
		No (3/16)	79.4			

(*N* = 95 patients).

The wild type is underlined.

Gene-gene interactions between the variants and survival or relapse were analyzed with multifactor dimensionality reduction. There was no statistically significant multi-gene interacting model for death or event.

## Discussion

This study is the first pharmacogenetic analysis of Korean pediatric ALL and shows different distribution of genetic polymorphisms and different prognostic significance in survival compared to Western Caucasians.

Targeted genes in our study were proved to play important roles in the treatment of ALL. Thus, such genes could influence complications and survival rates. However, most of the previous pharmacogenetic studies were conducted on Western populations. Rocha *et al.* conducted a pharmacogenetic study in 246 pediatric patients enrolled for St Jude Children's Research Hospital Total XIIIB study, and reported that *GSTM1* non-null genotype was associated with an increased risk of hematologic relapse, and *VDR* FokI T allele with CNS relapse in higher risk patients [11]. Reports of genetic polymorphisms on the genes involved in the folic acid cycle also showed clinical implications. Aplenc *et al.* analyzed samples of 520 patients in CCG-1891 which was the backbone of our institutional protocol, and reported that the C677T variant of *MTHFR* was statistically significantly associated with relapse [25]. In the study of Rocha *et al*, the homozygotes of 3 tandem repeats (3R/3R) in the polymorphic 28-base-pair region of *TYMS*, which is one of the catalytic enzymes in the folic acid cycle, was associated with greater risk of hematologic relapse due to the higher expression of TYMS than a 2R [11].

In additions to survival and relapse, genetic variations in the drug metabolisms are also known to influence treatment related toxicity. *TPMT* is one of the most well defined genotype among those. TPMT deficient individuals (*TPMT* variants) form higher concentrations of the thioguanine nucleotides and are more susceptible to acute thiopurine toxicity, such as myelosuppression, at standard doses of mercaptopurine [26], [27].

In this study, however, results were different from earlier studies in Western populations. There was no correlation between survival or relapse and genotypes known as to have clinical implications in the earlier studies. As for drug toxicity, no significant correlation was noted in the plausible toxicities with a certain polymorphism. We used dose percents of 6-MP and MTX as surrogate markers for toxicity. Our chemotherapy protocols adjusted the dose of 6-MP and MTX to reach a target absolute neutrophil count. In this study, the dose percent of MTX was significantly correlated with the dose percent of 6-MP (*R* ^2^ = 0.628, *P* = 0.00). This result was due to the fact that the administration and dose reduction of MTX were influenced by the same factors as myelosuppression and liver toxicity with 6-MP. There was one patient in this study who showed *TPMT* variant homozygous genotype, but he demonstrated mild toxicity, so that final dose percents of the patient were 62% in 6-MP and 85% in MTX. Although no significant difference in dose percents by each *TPMT* genotype was observed in our study, the median dose percent for *TPMT* wild type was higher than variant types in both drugs.

To interpret these discrepancies from Western pharmacogenetic studies, several factors could be considered. The dose modification of our protocol is one possible explanation. The dose of 6-MP was 75 mg/m^2^/d in the original CCG and St. Jude protocols, which was bigger than our dose of 50 mg/m^2^/d. In the report of the BFM group that used 50 mg/m^2^/d of 6-MP, there was no significant difference in toxicity between *TPMT* wild types and variants [28]. Because the dose and toxicities of 6-MP and MTX are tightly correlated in maintenance phase of the ALL treatment, it could be inferred that the higher dose might elevate risks of toxicity, and different doses of 6-MP could be an important factor in pharmacogenomic studies including 6-MP and MTX related genes. Therefore, different toxicity profiles and dose might also influence treatment outcome. The difference in the incidence of polymorphic allele from Caucasians is the other explanation for the discrepancies. In addition, there are many other genes which involve the metabolism of mercaptopurine, and synergistic epistatic interaction within these genes was recently reported as an influencing factor for hematological toxicity of mercaptopurine [29]. Dorababu *et al.* reported that epistatic interactions between the variations of *TPMT* (*3C, *12) and *ITPA* (rs1127354, rs8362) were associated with the 6-MP toxicity by multifactor dimensionality reduction analysis [29]. In our study, we analyzed gene-gene interactions with the same analysis. Multifactor dimensionality reduction is a nonparameric method which detects interactions by pooling multiple loci into high-risk and low-risk groups depending on whether they are more common in affected or in unaffected subjects and by reducing the dimensionality of the multilocus data to one dimension [30]. MDR is known to have reasonable power to identify interactions among two or more loci in relatively small samples [31]. However, there was no meaningful epistatic interactings in our study, which was different from Dorababu's study with Indian patients, and this difference might be also from ethnic differences and protocol differences.

Complex trait of toxicity manifestations is the other explanation for the discrepancies between pharmacogenetic studies. The impact of variant alleles might be modified by many factors, such as concurrent medications, diet, or other environmental factors. Those factors differ substantially between ethnic groups or countries. In addition, a relatively small number of event and toxicity cases limited the statistical power of our study to detect modest effects of variant genotypes on treatment outcome and toxicities.

Another issue which should be considered in pharmacogenetic studies is age. In children, specificity of drugs for individual enzymes and drug disposition may differ from adults and pharmacogenetic gene expression is not fully matured so that efficacy and safety can be variable and different [27], [32]. Among enzymes which were encoded by genes included in our study, TPMT activity in peripheral red blood cells was similar from neonates to adults [33], whereas CYP3A5 was lower in activity in the liver samples of CYP3A5*3 genotype variants [34]. Although thorough evaluation of metabolic differences by age is lacking due to difficulties in blood sampling and ethical problems, most of the pharmacogenetic studies in ALL were conducted in children, which allows relatively easy comparison with previous studies.

We analyzed clinical implications of *ITPA* polymorphism in this study. Like *TPMT*, *ITPA* shows genetically determined polymorphic activity, and patients who inherit non-functional alleles have been shown to be more sensitive to 6-MP [9]. The *ITPA* 94 C>A transversion causes an amino-acid change (P32T), reducing ITPA enzymatic activity to 25% in heterozygotes, and abolishing it in homozygous variants [10]. According to the St. Jude's study, *TPMT* genotype was a significant determinant of 6-MP and toxicity when 6-MP was not adjusted for *TPMT*. After adjustment for *TPMT*, however, the additional influence of *ITPA* on 6-MP toxicity emerged [10]. Stocco *et al.* reported that the cumulative incidence of febrile neutropenia during treatment of 6-MP individualized for *TPMT* was significantly greater among patients with *ITPA* variant alleles. Those toxicities could be due to the higher concentration of methylated thiopurine nucleotides, which had cytotoxic properties.

In our study, there was no difference in the cumulative incidence of grade 3, 4 febrile neutropenia according to *ITPA* genotypes. In addition, no statistical difference in dose percents by each *ITPA* genotype was observed, and no reduction trend in *ITPA* variants was observed. Our protocol was not individualized for *TPMT* genotypes, so that the influence of *ITPA* variant on toxicity did not emerge.

One novel finding of our study was a possible association between survival rate and *ITPA* polymorphism. In previous studies, *ITPA* genotype significantly influenced the risk of fever and neutropenia, but this did not influence survival rates. Stocco *et al.* postulated that the *ITPA* polymorphism significantly influenced the risk of toxicity without influencing efficacy because they had immediately treated febrile neutropenia with antibiotics [10]. However, in our study, event free survival rate was significantly lower in the patients with *ITPA* 94 AC/AA variant genotypes in the univariate and multivariate analyses. A total of 5 patients relapsed in the *ITPA* variant group (BM = 1, CNS = 1, testis = 1, BM and CNS = 1, and BM and testis = 1), and 2 patients among them died due to transplantation-related complications (veno-occlusive disease, cytomegalovirus pneumonia). Four relapsed patients showed *TPMT* wild type, and the other one showed TPMT *1/*3C alleles. Because the incidence of febrile neutropenia or sepsis was not higher in *ITPA* variants and death did not occur during the courses of mercaptorpurine or methotrexate, the lower survival rate in the variant group might not be influenced by accumulated toxic metabolites, but by other factors.

The plausible explanation of this result was that other genetic polymorphisms which were linked with *ITPA* 94 C>A (rs1127354) might have a clinical impact. Recently, one genome-wide association study (GWAS) identified that four SNPs (rs11697186, rs6139030, rs1127354 and rs13830) located on *DDRGK1* and *ITPA* genes on chromosome 20 showed a strong linkage disequilibrium (LD) (r^2^>0.86) within the 22.7 kb [35]. In previous studies, *ITPA* 94 C>A (rs1127354) was known as a high risk factor for ribavirin induced toxicity in patients with chronic hepatitis C. In this recent GWAS study, Tanaka *et al.* revealed that SNPs on *DDRGK1* and *ITPA* both had strong associations with hematologic toxicity in response to pegylated interferon and ribavirin therapy for chronic hepatitis C. DDRGK1 (DDRGK domain-containing protein 1) is a novel C53/LZAP-interacting protein. C53/LZAP is a putative tumor suppressor that plays important roles in multiple cell signaling pathways, including DNA damage response and NF-kappaB signaling [36]. However, it remains largely unknown how the function of *DDRGK1* variants is regulated. Likewise in this recent GWAS study, if there was other genetic variant which influenced event free survival in our study, the variants of *DDRGK1*, which is a neighbor gene of *ITPA*, could be possible candidates. Further studies are required to elucidate the possible association between *DDRGK1* variants and clinical outcomes of ALL.

In addition, this different result from Caucasians might be influenced by different allele frequency. Allele frequency for *ITPA* 94 C>A is known to be 19% in the Asian population [37]. However, the allele frequency of this polymorphism shows significant inter-ethnic variability, ranging from 1–2% in Hispanic populations to 5–7% in Caucasian population [37]. Interestingly, according to the report of Marsh *et al.*, this variant allele shows almost a reversal in allele frequencies when compared with *TPMT* [37]. They proposed that Asians who have a low frequency of *TPMT* variant alleles may be more susceptible to the influence related to *ITPA* variant alleles, while Caucasian and Hispanic patient population may be more easily related to *TPMT*. This predominance might reveal this influence of *ITPA* on survival. Our result needs further confirmation in a larger population because besides *ITPA* polymorphisms, other factors such as other drug-metabolizing enzymes, heterogeneity in genetic background and clinical characteristics can influence response to treatment.

In this study, ethnic difference was significant in the incidence of genetic polymorphisms between Koreans and Western Caucasians. Ethnic differences in survival of pediatric ALL have been reported in many studies, and the difference is thought to be caused by multiple factors, including different genetic polymorphic distributions [38], [39]. Ethnic difference in our study became evident when we compared our data with Japanese data. Although there was also some difference in the variant frequencies with normal Japanese, those were noted in fewer loci than the result with Caucasians, and Japanese data was from normal population so that this might influenced these discrepancies.

This study has limitations, including the limited number of loci, modest numbers of patients in each variant, and the lack of information about various toxicities. To overcome this limitation of candidate polymorphism approach, a genome-wide study identifying disease-related genetic polymorphisms has been conducted recently with thousands of population scale. However, for analysis on a large scale, it is useful to effectively screen SNPs using data already reported. Those results provide useful information on candidate SNPs for pharmacogenetic surveillance. Furthermore, in most genome-wide studies, samples from Japan and China were used as East Asian references. This study is the first pharmacogenetic study in Korean pediatric ALL, so that more extensive studies regarding the pharmacogenetics of Asians can be conducted in the future based on this report.
